# Research on Cold Roll Forming Process of Strips for Truss Rods for Space Construction

**DOI:** 10.3390/ma16247608

**Published:** 2023-12-12

**Authors:** Xingwen Yang, Jingtao Han, Ruilong Lu

**Affiliations:** 1 School of Materials Science and Engineering, University of Science and Technology Beijing, Beijing 100083, China; 2 Industrial Training Center, Zhongyuan University of Technology, Zhengzhou 451191, China; 3 Guangzhou Sino Precision Steel Tube Industry Research Institute Co., Ltd., Guangzhou 511300, China

**Keywords:** truss rods, cold roll forming process, roll gap, roll spacing, concentration of strain, tearing crack

## Abstract

In this paper, a new technology for on-orbit cold forming of space truss rods is proposed. For the cold roll forming process of asymmetric cross sections of thin strips, the effects of roll gap and roll spacing on the forming of asymmetric cross sections of strips were investigated using ABAQUS simulation + experiments. The study shows the following. When forming a strip with a specific asymmetric cross section, the stresses are mainly concentrated in corners 2/4/6, with the largest strain value in corner 2. With increasing forming passes, when the roll gap is 0.3 mm, the maximum equivalent strain values are 0.09, 0.24, 0.64 sequentially. Roll gaps of 0.4 mm and 0.5 mm equivalent strain change amplitude are relatively similar, and their maximum equivalent strain values are approximately 0.07,0.15, 0.44. From the analysis of the stress–strain history of the characteristic nodes in corners 2/4/6, it can be seen that the stress and strain changes in the deformation process mainly occur at the moment of interaction between the upper and lower rollers, where the stress type of node 55786 shows two tensile types and one compressive type, the stress type of nodes 48594 and 15928 shows two compressive and one tensile type, and the strain of the three nodes is in accordance with the characteristics of plane strain. When the roll gap is about 0.4 mm, the forming of the strip is relatively good. With increased roll spacing, the strip in the longitudinal stress peak through the rollers shows a small incremental trend, but the peak stresses are 380 Mpa or so. When the roll spacing is 120 mm, the longitudinal strain fluctuation of the strip is the most serious, followed by the roll spacing at 100 mm, and the minimum at 140 mm. Combined with the fluctuation in strip edges under different roll spacings, manufacturing cost and volume and other factors, a roll spacing of 100 mm is more reasonable. It is experimentally verified that when the roll gap is 0.4 mm and the roll spacing is 100 mm, the strip is successfully prepared in accordance with the cross-section requirements. When the rolling gap is 0.3 mm, due to stress–strain concentration, the strip is prone to edge waves in forming. The top of corner 2 of the flange triangular region is susceptible to intermittent tear defects, and the crack extension mechanism is mainly based on the cleavage fracture + ductile fracture.

## 1. Introduction

An important part of spacecraft structure, the space truss is mainly constructed by connecting a certain number of one-dimensional rods in three-dimensional space in a certain direction, and has been widely used in constructing deep space exploration bases and expanding the functions of space stations [1]. However, in current spacecraft, space truss structures are typically in the order of 10–1000 m in size for on-orbit operation [2,3]. Future space exploration will require large, lightweight, high-performing and cost-effective space trusses, such as in-orbit service platforms, which will reach geometric dimensions of 0.1–10 km when they are completed [4,5]. Obviously, the existing methods of deployment and assembly on orbit are very limited [6,7], and new ways of building are urgently needed. The use of various space manufacturing technologies, including on-orbit additive manufacturing [8,9] and on-orbit welding [10,11], to produce the rods is one of the effective ways to solve the bottleneck problem of large space trusses on orbit. In this paper, an on-orbit cold forming manufacturing technology is proposed for the production of space truss rods, which mainly uses small cold roll forming machines to coil the raw material into rods to realize on-orbit manufacturing. The method is simple in principle, easy to implement, and does not need to take into account a number of complex problems, such as the suspension of molten droplets in microgravity and solidification in space [2], and is expected to provide a new way of manufacturing truss rods on orbit.

**Figure 1:**
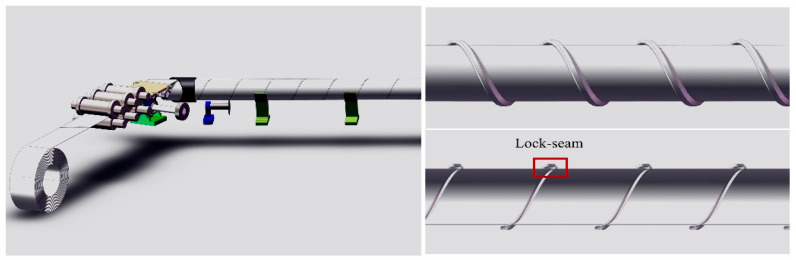
Schematic diagram of the production of truss rods by means of cold roll forming technology.

**Figure 2:**
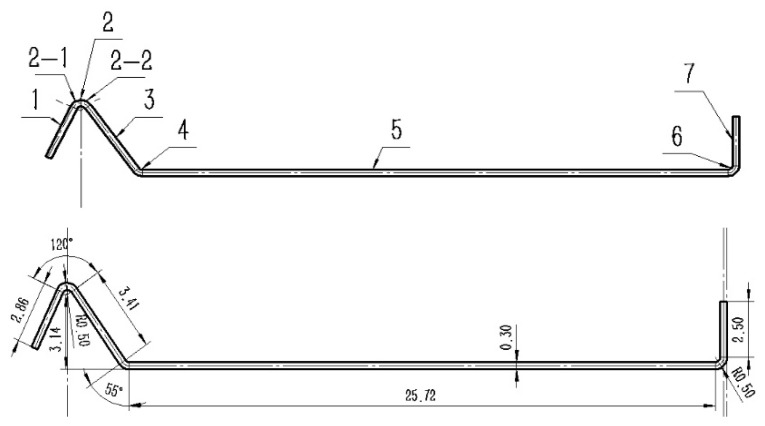
Partitioning and dimensions of the individual parts of the strip with a specific asymmetric cross section formed in the cold roll forming process. 1—partition 1, 2—partition 2 (Includes partitions 2-1 and 2-2), 3—partition 3, 4—partition 4, 5—partition 5, 6—partition 6, 7—partition 7.

The principle of using cold forming manufacturing technology to produce truss rods on orbit proposed in this paper is as follows. First, the strip in coils is passed through a small cold forming machine to form a strip with a specific cross section. Next, the strip, with a specific cross section, will be subjected to spiral molds and pressure wheels to achieve spiral bending, locking seam, and compaction, ultimately allowing for the manufacture of truss rods of any desired length. The principle is illustrated in Figure 1. In this technology, the initial cold roll forming of the strip to a specific cross section using multiple sets of rolls is an incredibly important step that has a direct impact on the subsequent success of the strip spiral bending and locking seam process. The partitioning and dimensions of the asymmetrical cross-section strip designed for this subject are shown in Figure 2. The investigation of the cold roll forming process mainly focuses on the production of cold-formed components with symmetrical cross sections [12]. These include items like hollow square tubes [13], U-shaped steels [14,15], C-beams [16,17], V-beams [18], W-section plates [19], hat-shaped plates [20,21], and corrugated plates [22,23]. Safdarian et al. [24] studied the formation process of square tubes by cold roll forming and found that the longitudinal strain of the strip edge was not substantially affected by the friction between the strip and the roll or by the rolling speed. Poursafar et al. [19] investigated the effect of each anisotropy of material plasticity and angular increment or pattern, strip width on springback, and longitudinal bending during forming in W-profile sheets. Najafabadi et al. [25] investigated the edge wrinkling mechanism during cold rolling of wide continuous U profiles. For asymmetric cross-section strips, due to the complex roll hole design and the need to consider force balance deformation and other issues specific to asymmetric cross sections, there has been limited research into the related aspects of cold roll forming. Wang et al. [26] investigated the effect of roll gap, friction coefficient, roll diameter increment and linear speed on the maximum longitudinal strain at the strip edge when forming asymmetric and deep complex cross sections, which mentioned that the effect of roll gap was very important, but did not conduct much in-depth research. The present investigation shows that strip thicknesses for cold roll forming are generally greater than 1 mm; however, this paper proposes a process where the strip thickness used is in the range of 0.3–0.5 mm for rod forming. When cold roll forming thin strips, the influence of the roll gap on the cold roll forming of the strip becomes more significant. Additionally, the design of thin strip cold roll forming equipment must also consider the roll spacing as an important parameter due to limitations on the equipment size imposed by the space station. Therefore, in order to form the target cross section of the strip, we developed an asymmetric cross-section strip roll gap self-adjusting equipment, focusing on the study of the thin strip in the process of the cold roll forming roll gap and roll spacing on the asymmetric cross section of the strip forming effect. ABAQUS numerical simulation was used to study the transverse stress and strain on the asymmetric cross section of a strip in the cold roll forming process, the change in stress and strain history in the corner regions of the cross section, the dimensional accuracy of the strip cross section, and the influence of the roll spacings on the longitudinal stress and strain of the strip to find the appropriate process parameters and to conduct experiments to verify the results. At the same time, the metallographic microstructure of corner 2 in the flange triangular part of the strip in the experiment was observed, and the cracking defects appearing in the strip during the forming process were analyzed using a scanning electron microscope, which revealed the reasons for the cracking of thin strips and their expansion mechanism. In this way, it provides a certain reference for the cold roll forming preparation of thin strips with an asymmetric cross section and contributes to the realization of space truss rods from strips to rods in on-orbit cold forming manufacturing technology.

## 2. Experimental Materials and Methods

### 2.1. Material Properties

As the cold roll forming process involves small diameter and large bending deformation of the strip, there are high demands on the plastic toughness of the material. A cold-rolled strip of Q195 galvanized steel was used as the test specimen, and its mechanical properties and chemical composition are shown in Table 1.

The strip is 0.3 mm thick and 36 mm wide. The strip is subjected to a longitudinal tensile test and the engineering stress–strain curve obtained is converted to a true stress–strain curve. After the strip enters necking, the Holloman hardening method is used to obtain σ = 7227·ε^0.183^ based on the relevant data, the ABAQUS (Version 2018) model showed that the Q195 strip has a density of 7.85 g/cm^3^, an elastic modulus E of 2.06 × 10^5^ MPa and a Poisson ratio of 0.3.

### 2.2. Flower and Roll Design

**Figure 3:**
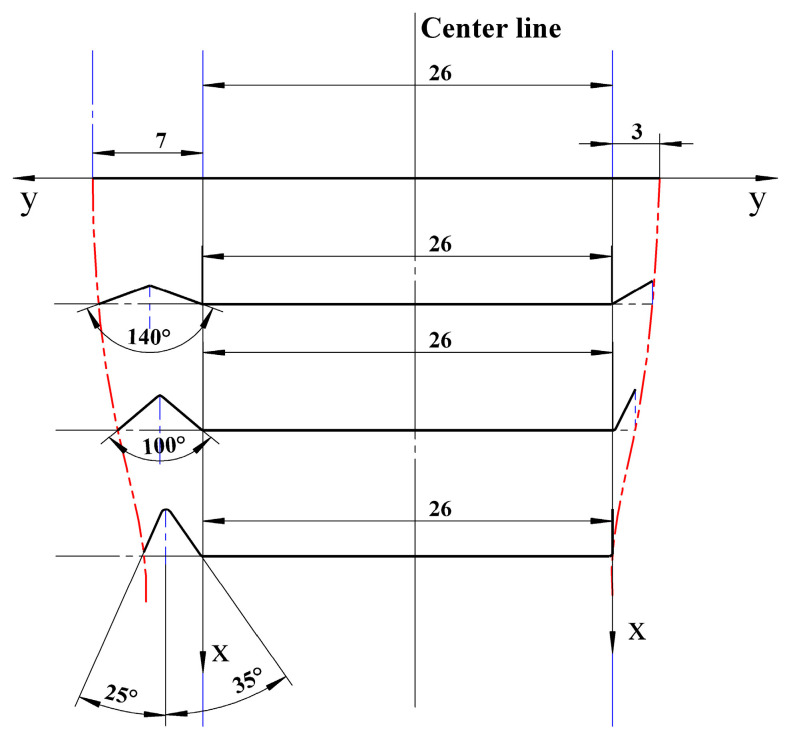
Flower diagram of a strip with the target cross section according to the principle of cubic curves of the horizontal projected trajectory of the end of the vertical edge.

As the equipment will be used in orbit on a space station, it needs to be miniaturized. On the basis of the dimensions of the strip in the target cross section, the number of forming passes is calculated using the method of calculation of the number of forming passes to be 4. The first pass is precompression, and deformation is mainly concentrated in passes 2–4. The distribution of the roll forming channel angle follows the principle of the cubic curve of the horizontal projection trajectory of the end of the vertical edge. The flower pattern of the designed target cross-section strip is shown in Figure 3. As the profile of the target cross section is asymmetrical, the angle of each set of rollers is designed by taking into account the variation index using Equation $\cos\theta_{i}=1+(1-\cos\theta_{\circ })[2(\frac{i}{N}{)}^{3+\kappa }-3(\frac{i}{N}{)}^{2-\kappa }]$. The bending angle distribution of the rollers for each pass is shown in Table 2. The cubic curve equation obtained for the left side of the target cross-section strip (the flange triangular region) is:

(1) $$f(x)=8.464e^{-5}x^{3}-0.008047x^{2}+0.005208x+7$$

**Figure 4:**
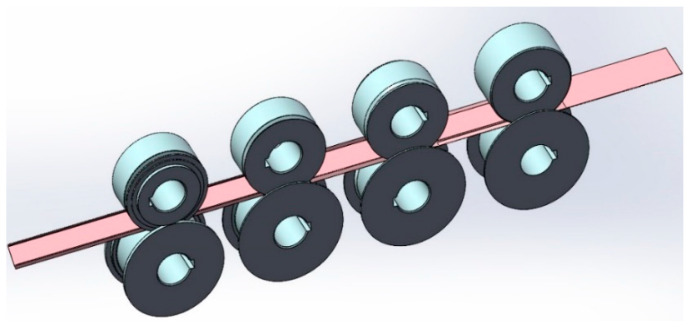
Schematic of the designed 3D model for cold roll forming of strips.

The cubic curve equation obtained for the right side of the target cross-section strip (right-angled region of the flange) is:

(2) $$f(x)=9.766e^{-5}x^{3}-0.007422x^{2}-0.003125x+3$$

where x=8(n−1), n is the number of forming passes (since the first pass is a precompression-guided pass, the effective passes start from the 2nd pass). According to the roll bending angle distribution Table 2 for the roll set design, taking into account the volume factor, the roll design of its base circle diameter of 70 mm, the design of the strip cold roll forming three-dimensional model shown in Figure 4.

### 2.3. Experimental Scheme and Finite Element Modeling Process

In this paper, we mainly study the effect of different roll gaps and roll spacings on the cold roll forming process of thin strips with asymmetric cross sections and design a simulation experiment scheme, as shown in Table 3. The reasonableness of the simulation results and the accuracy of the rolled products are then verified experimentally.

**Figure 5:**
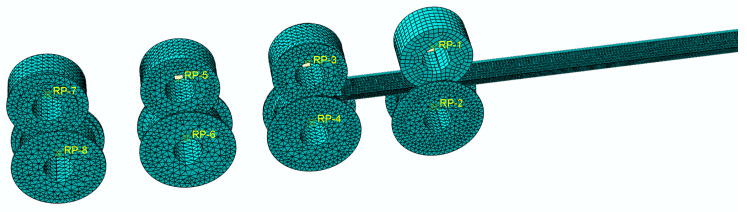
Cold roll forming model of strip with meshed view.

**Figure 6:**
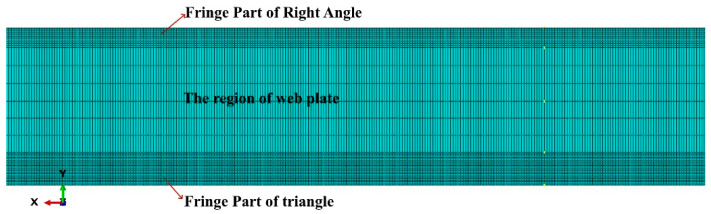
Schematic diagram of the subdivided strip mesh.

The strip cold roll forming process was modeled using ABAQUS as follows. In the cold roll forming process by means of four sets of forming rolls, the first set of rollers are prepress rollers with no significant deformation, so the model is built by biting into the strip directly from the second set of rollers adopting the ABAQUS/Explicit dynamic analysis model. A deformable body cell of cell type C3D8R (eight-node linear hexahedral cell, reduced integration, hourglass control) is used for the strip to accurately reflect the deformation process in each part of the strip. Hourglass control mode adopts stiffness hourglass control, because for plastic bending problems, better calculation results can be obtained by using stiffness-based hourglass control. Meanwhile, a reasonable mesh refinement is applied to the strip model. The artificial strain energy (ALLAE) of the strip after forming is found to be not more than 1.5% of the internal energy for the whole model (ALLIE) in numerical simulation, which indicates that the hourglass is controllable and the calculation results in this mode are accurate. The roller sets are set as discrete rigid bodies with unit-type C3D10M (ten-node modified quadratic tetrahedral unit). Construct the model using SolidWorks and import it to ABAQUS. The beveled section of the rollers is stitched to aid in the meshing of the roller sets. To investigate the impact of the roll gap, the roll gap was adjusted to 0.3 mm, 0.4 mm and 0.5 mm, respectively. The roller sets are equally spaced and modeled with roll spacing of 100 mm/120 mm/140 mm, respectively. Four sets of rollers (eight rollers) are rigidly fixed and the relative speed of the upper and lower rollers is 5 r/s. In the interaction module, the generic contact algorithm is selected. The contact properties of the interaction are set for normal behavior and tangential behavior. The friction formula for tangential behavior is the penalty formula, which is applicable to most metal forming problems, the generic coulomb friction is selected, and the friction coefficient is set to 0.2. The pressure overclosure is set to hard contact for normal behavior, and the constraint enforcement is set to default mode. The length of raw strip is 800 mm. To precisely depict the distortion of the flange section in the strip’s cross section, the mesh of the flange fragment requires refinement for mesh sizes between 0.15 mm and 0.375 mm. The deformation in the central section is minimal and the mesh is coarser, with a size of 3.9 mm. The meshed cold roll forming model of the strip is illustrated in Figure 5. The subdivided strip mesh is shown schematically in Figure 6.

## 3. Results and Discussion

### 3.1. Influence of Roll Gap on Cold Roll Forming of Strip

#### 3.1.1. Influence of Different Roll Gap on Strip Stress–Strain during Each Forming Pass

**Figure 7:**
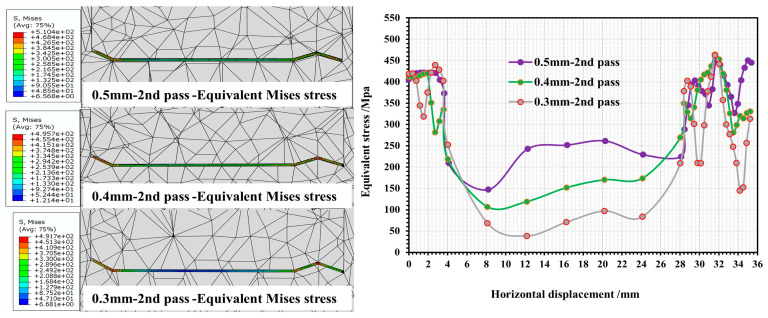

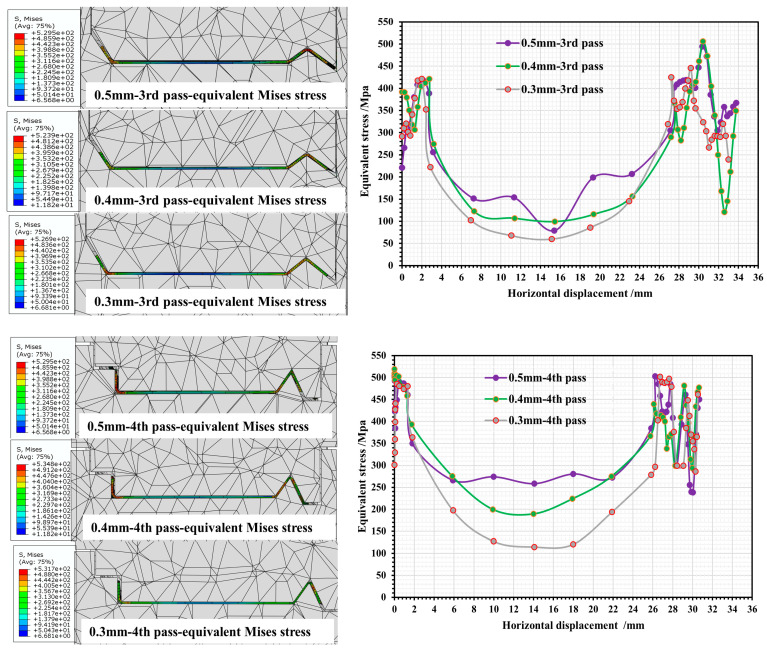
Stress variation in strip cross section at 2nd/3rd/4th pass with different roll gaps.

**Figure 8:**
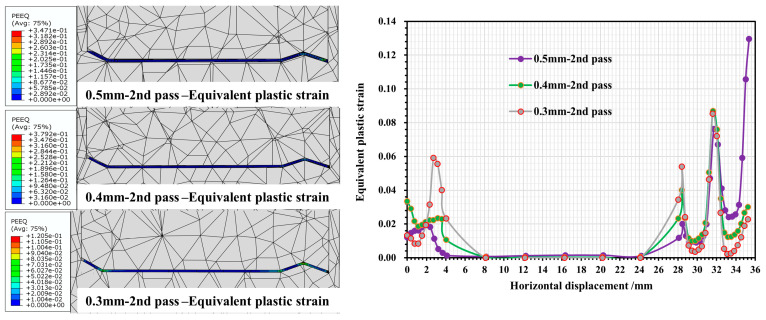

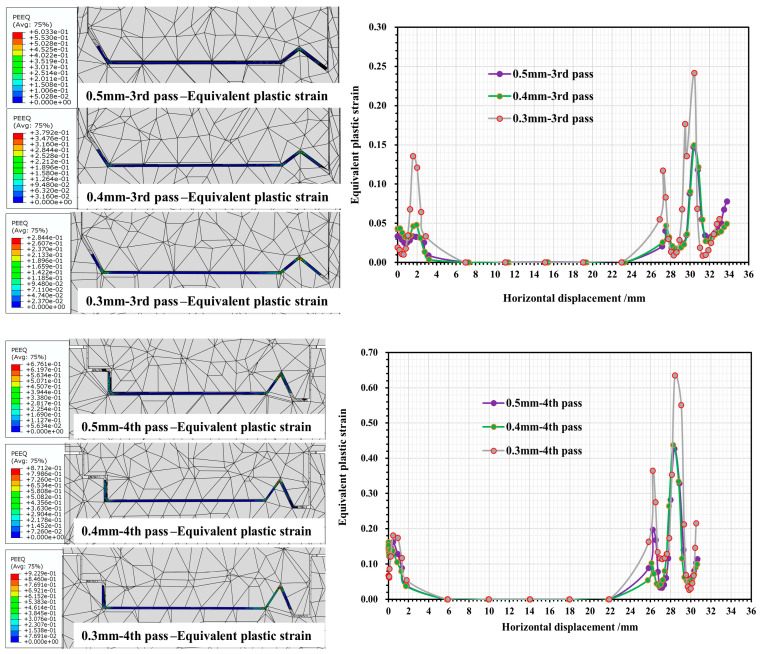
Strain variation in strip cross section at 2nd/3rd/4th pass with different roll gaps.

When the roll spacing is 120 mm, the stress and strain of each forming pass of the strip under different roll gaps (0.3 mm/0.4 mm/0.5 mm) are shown in Figure 7 and Figure 8.

As can be seen in Figure 7, during the forming process of each pass, the peak equivalent stresses of the strip under different roll gaps are mainly concentrated in the regions of corners 2, 4 and 6. With the increase in the number of forming passes, there is a slight incremental trend in the stress of the above parts, and the stress value fluctuates around 403–515 MPa. In passes 2 and 3, the highest equivalent force is concentrated in corner 2 at the flange triangular region of the strip. However, when it comes to pass 4, the maximum equivalent force value begins to move from corner 2 (located at the top of flange triangle region of the strip) to corner 4 (situated at the bottom of the flange triangle region). Changing the roll gap value has no significant effect on the equivalent force value at the corners of the curve. However, for web plate 5 in the middle of the strip section, the equivalent stress value in this region is 60–280 MPa, and with the reduction in roll gap, the stress in this part is relatively low, showing a gentle fluctuation, and can be analyzed from the value of the force as mainly elastic stress. As can be seen in Figure 8, there are significant strain peaks in the region of corners 2, 4 and 6 where the equivalent stress values are greater. In corners 2 and 4 of the flange triangular region of the strip, the effect of the roll gap on the equivalent strain is apparent. When the roll gap is 0.3 mm, the maximum equivalent strain values in corner 2 increase with the number of passes, reaching 0.09, 0.24 and 0.64 in succession. The strain values at corner 4 near the bottom of the triangular region are significantly higher than the corresponding values, with roll gaps of 0.4 mm and 0.5 mm. In order, the equivalent strain values are 0.05, 0.12, and 0.39. The equivalent variation in roll gap of 0.4 mm/0.5 mm is relatively similar, and for corner 2, its equivalent variation is about 0.07/0.15/0.44, but corresponding to corner 4, when the roll gap is 0.4 mm, its equivalent variation is the lowest, only 0.2. For corner 6 of the flange right-angled region of the strip, in the second and third passes, the equivalent strain value is larger when the roll gap is 0.3 mm, but upon entering the fourth pass, the final equivalent strain values of each roll gap tend to be the same, which is approximately 0.2. The equivalent strain values in web plate 5 of the strip are all zero, which is also consistent with the equivalent stress values in Figure 7, indicating that the web plate region is mainly dominated by elastic strain and no plastic deformation occurs.

From the above analysis, it can be seen that the strip cross section in the deformation process, mainly for the strip cross section of the deformation of the corners, in the corners, since the bending angle of corner 2 at the flange triangular region of the strip is the smallest, the stress–strain value at this part is also the largest. As the roll gap decreases and the number of passes increases, all corner regions are subjected to varying degrees of plastic stress, resulting in an increase in strain as the bending angle of the corner region decreases. The strain surge occurs mainly in the fourth pass, and excessive strain values can lead to localized thinning of the strip in these regions, or even to wrinkling and cracking. When the gap between rolls is relatively large, the strip can obtain a larger deformation space during deformation, which can effectively alleviate the strain concentration phenomenon, but may have a greater effect on strip asymmetric cross-sectional forming accuracy.

#### 3.1.2. Analysis of Changes in Stress–Strain History at Bend Corners

**Figure 9:**
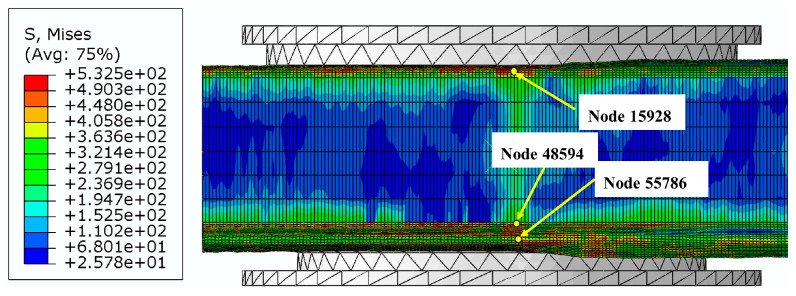
Schematic diagram of the location of the feature nodes in the selected corners.

**Figure 10:**
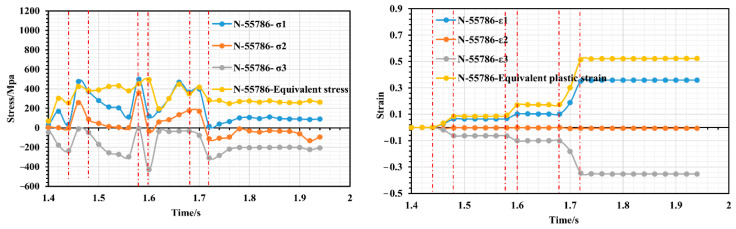
Stress–strain history variation of node 55786.

**Figure 11:**
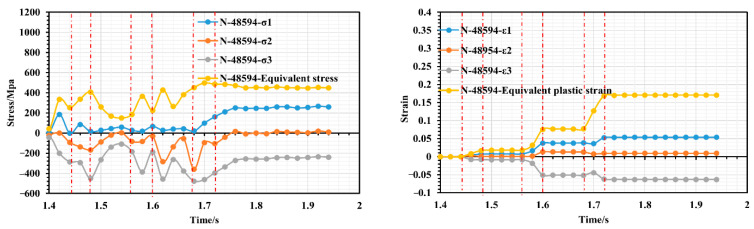
Stress–strain history variation of node 48594.

**Figure 12:**
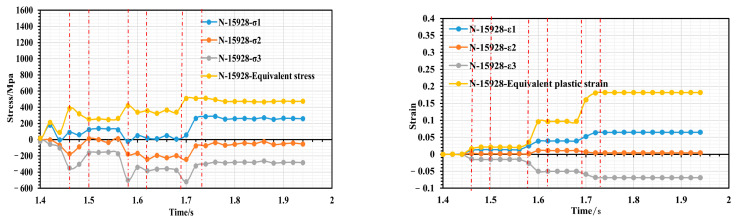
Stress–strain history variation of node 15928.

Taking the example of a roll gap of 0.4 mm, we selected the nodes with significant features, namely, 55786, 48594, and 15928, from the fourth forming roll located near corners 2, 4 and 6 to examine the changes in stress–strain history. Figure 9 shows a schematic diagram of the location of the selected characteristic nodes, Figure 10 shows the variation in stress–strain history of node 55786 (the top of corner 2), Figure 11 shows the variation in stress–strain history of node 48594 (inner side edge of corner 4 at the bottom of the flange triangular region), and Figure 12 shows the variation in stress–strain history of node 15928 (inner side edge of corner 6 at the flange right-angled region).

As depicted in Figure 10, the strip initiates entering the second set of rollers at t = 1.44 s and fully exits it at t = 1.48 s. During this period, a slight peak in the equivalent force is observed. The strip takes 1.48–1.58 s to travel between the second and third roller groups. During this time, the equivalent strain remains constant; however, tension between the two roller groups causes internal stress to fluctuate, with a predominance of elastic stress. At 1.58 s, the strip enters the third roll group. Due to the narrowing of the strip’s cross section, it quickly experiences peak stress and further deformation. The equivalent stress continues to increase; however, as the increase in bending angle is small at this stage of deformation, the increase in equivalent plastic strain is not significant and only increases from 0.1 to 0.18. At 1.66 s into the process, the strip begins to enter the fourth roll group and as the cross section of the strip narrows, node 55786 at the inner edge of the flange triangle region quickly moves to the top of corner 2, resulting in a small peak of stress just before entering the fourth roller group. Upon entering the fourth roll group, the node experiences additional plastic tensile deformation at the top of corner 2. This results in a rapid increase in the equivalent plastic strain from 0.18 to 0.52. Although the bend angle increment is basically the same, the bend angle at this point is significantly smaller, and both sides of the triangle region have a large tensile effect on the top of corner 2 during deformation, which ultimately leads to greater strain at this point, making it the most susceptible to defects in the forming process. Overall, it appears that unit node 55786 has a step increase in strain during the cold roll forming process. It only has a strain surge just between the roller sets entering, after which it remains in a constant strain state for the duration of the movement of the two roller sets. Also, based on Figure 10, it can be concluded that whenever the strip passes through the rollers, node 55786 experiences a three-way stress state consisting of two tensile stresses and one compressive stress. The strain state is plane strain. Since node 55786 is situated on the outer surface of corner 2, this corresponds with the theoretical mechanical analysis at that particular location. As shown in Figure 11 and Figure 12, the stress–strain histories of nodes 48594 and 15928 are similar, the two nodes are located in the inside of the bending corners, and in the process of deformation, with the increase in the number of forming passes, their equivalent stresses show an increasing trend during the action of each group of rollers. However, during the travel of the roller groups, node 48594 on the strip experiences more violent stress fluctuations; this is likely due to its location at the junction of the edge of the flange triangular region and the web plate, which causes it to undergo bidirectional bending deformation, and it is therefore subjected to more complex stresses than the right-angled edge of the flange, which is subjected to unilateral bending. In total, the maximum equivalent strain of both nodes is below 0.18, indicating a small strain value and relatively smooth deformation; the stress state of the two nodes also shows three-way stress, but its type is two compressions and one tension, and the strain state also basically corresponds to the characteristics of plane strain.

#### 3.1.3. Influence of Different Roll Gaps on the Asymmetric Cross-Section Dimensional Accuracy of Strips

**Figure 13:**
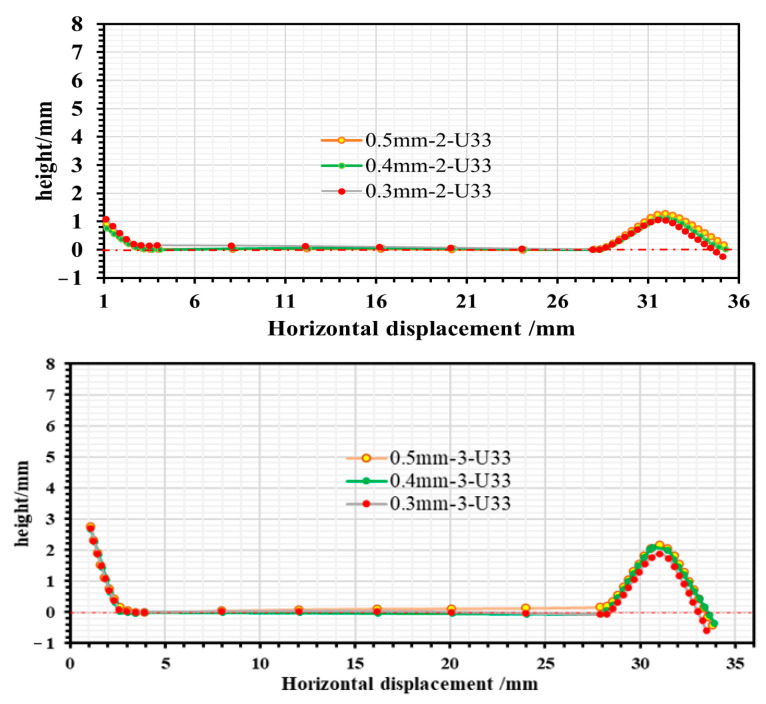

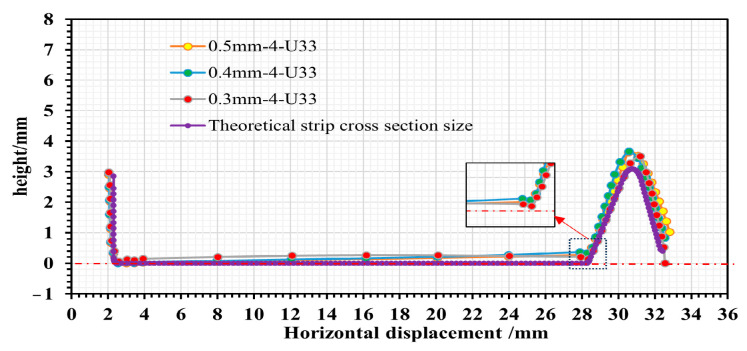
Deformation of strip asymmetric cross-sectional dimensions during the 2nd/3rd/4th passes under different roll gaps.

From Figure 13, it can be seen that the roll gap has little effect on the deformed dimensions of the right-angled part of the flange of the strip asymmetric cross section, but it has a significant effect on the triangular part of the flange. As the roll gap decreases, the triangular part approaches the desired size. However, as the number of forming passes increases, the cumulative strain of the triangular part becomes greater than that of the right-angled part. This results in deformation phenomena such as warping of the triangular part under stress after forming. When the roll gap is 0.3 mm, it becomes comparable to the thickness of the strip due to the rebound of the bent corner regions, which results in an excessive squeezing of the triangular part during the fourth pass forming. The result is an excessive elongation of the flange triangular region of the strip and a concave phenomenon in corner 4 connecting the flange triangular region and the web plate. The thinning phenomenon occurs in this region, indicating that the strain in this location ought to be greater, which can be verified from Figure 8. Therefore, taking into account factors such as the stress–strain of the strip cross section and the accuracy of the strip dimensions, the roll gap of 0.4 mm is relatively good.

### 3.2. Effect of Roll Spacing on the Longitudinal Stress–Strain of Strips

**Figure 14:**
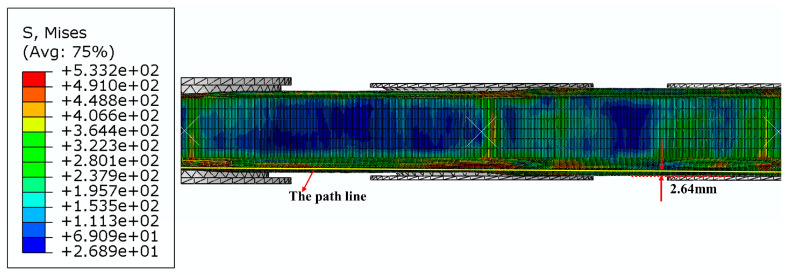
Schematic of the path in the strip at 2.64 mm from the outer edge of the flange triangular part.

**Figure 15:**
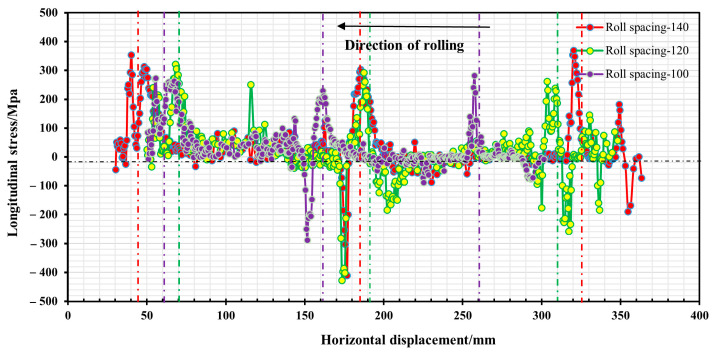
Longitudinal stress distribution in the strip at 2.64 mm from the outer edge of the flange triangular part.

**Figure 16:**
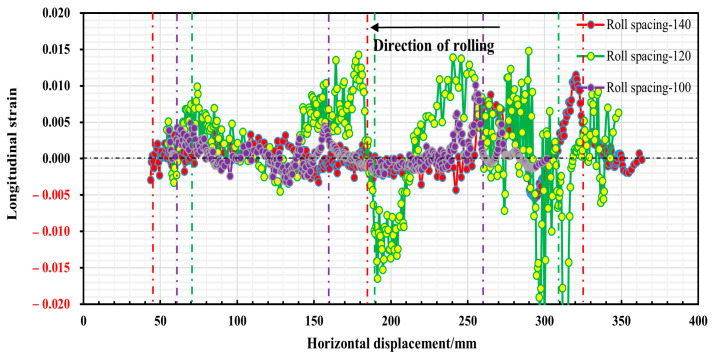
Longitudinal strain distribution in the strip at 2.64 mm from the outer edge of the flange triangular part.

**Figure 17:**
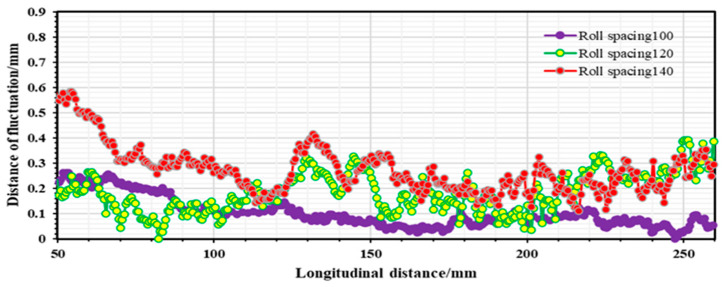
Fluctuations in the edge wave at the edge of the flange triangular part (i.e., at 0 mm).

When the roll gap was 0.4 mm, the changes in longitudinal stress and strain in the strip after the second, third and fourth forming passes during the strip forming process were investigated when the strip flange triangular part is 2.64 mm away from the outer edge, and the roll spacing is 100 mm, 120 mm and 140 mm, respectively. The established paths are shown in Figure 14, together with the stress and strain distributions along the paths shown in Figure 15 and Figure 16. In addition, strip edge wave fluctuation diagrams at the edge of the flange triangular part (i.e., at 0 mm) were also determined, as shown in Figure 17.

It is evident from Figure 15 that the longitudinal stresses within the strip during the forming process are predominantly tensile stresses. However, a fleeting longitudinal compressive stress is observed when the strip departs from the roll group in the third pass. With an increase in roll spacing, there is a slight incremental trend observed in the peak stress of the strip while passing through each roller. The peak stress measures around 380 Mpa, suggesting that the longitudinal stress of the strip is predominantly elastic stress. It can be seen from Figure 16 that the peak longitudinal strain at different roll spacings shows a decreasing trend as the number of passes increases. However, the longitudinal strain fluctuation is greatest when the roll spacing is 120 mm. As the number of passes increases, especially after the fourth pass, the longitudinal strain value for a roll spacing of 140 mm (peak strain of 0.002) is smaller than the longitudinal strain value for a roll spacing of 100 mm (peak strain of 0.004). It is shown that when the roll spacing is sufficiently large, it is possible to effectively reduce longitudinal strain. However, as roll spacing increases, it also leads to greater longitudinal stress, and this increase can result in strip rebound and exacerbate the strip’s fluctuation phenomenon, ultimately aggravating the edge wave phenomenon of the strip. Figure 17 illustrates the strip fluctuation of the edge wave at the edge of the flange triangular part t (i.e., at 0 mm). As observed, the edge wave phenomenon of the strip becomes more serious with an increase in roll spacing. At a roll spacing of 140 mm, the edge wave fluctuates between 0–0.5 mm, at 120 mm, it fluctuates between 0–0.35 mm, and at 100 mm, the edge wave of the strip is at its smallest, with a fluctuation of 0–0.26 mm. Furthermore, augmenting the roll spacing will result in increased volume of the forming roll set equipment and manufacturing costs. Taking into account multiple factors, the roll spacing of 100 mm appears to be the most suitable option.

### 3.3. Experimental Validation and Analysis of Defects in Strip Cold Roll Forming Process

**Figure 18:**
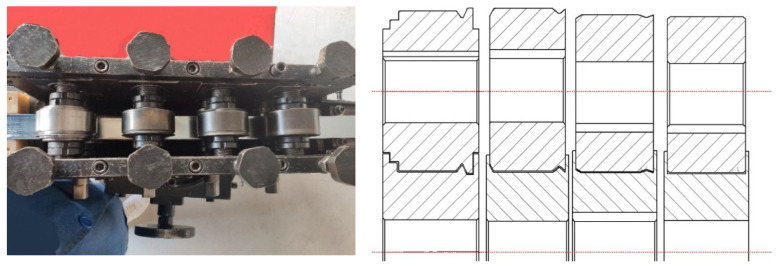
Preparation of cold forming roll set equipment with 100 mm roll spacing and designed roll set cross section.

According to the simulation results, the cold forming equipment was prepared with a roll base diameter of 70 mm, roll spacings of 100 mm and roll gaps with a self-correcting function. The physical equipment is shown in Figure 18.

**Figure 19:**
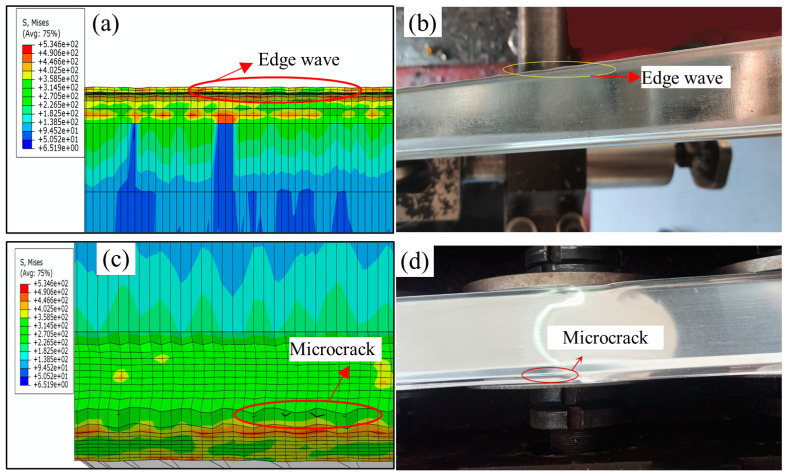
Simulation and experimental diagrams of the phenomenon of edge wave and tearing at the top of corner 2 during strip forming. (**a**) Edge wave phenomenon in simulation… (**b**) Edge wave phenomenon during strip forming (**c**) Tearing at the top of corner 2 in simulation (**d**) Tearing at the top of corner 2 during strip forming.

**Figure 20:**
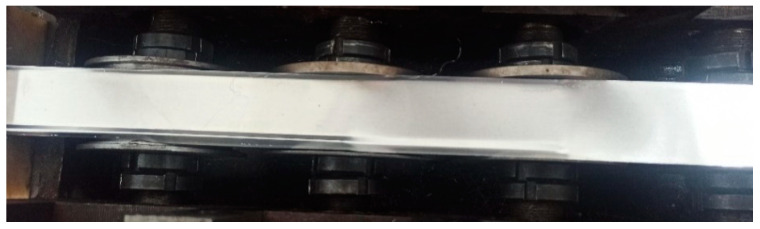
Surface quality topography of formed strip with roll gap of 0.4 mm.

**Figure 21:**
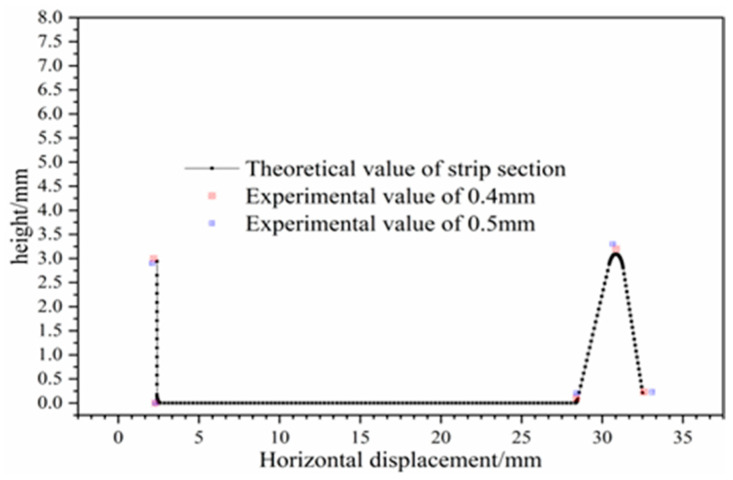
Test value of strip cross section at roll gap of 0.4 mm/0.5 mm.

During the simulation, the two main types of defects that appeared during strip forming were edge waves and intermittent crack lines that appeared at the top of corner 2, as shown in Figure 19. This was also verified in subsequent experiments. The appearance of these two types of defects is mainly due to the roll gap being too small, so that the strip in the passing roller groups surge in strain, and ultimately lead to defects; the above defects are generated in the roll gap of 0.3 mm when appearing. When the roller gap was increased to 0.35 mm, the experiment showed that the strip edge wave phenomenon almost disappeared, but there were still microcracks at the top of corner 2. When the roll gap increased to 0.4 mm, the surface quality of the strip was good, and the physical object of the strip formed in each pass is shown in Figure 20. By measuring the cross-sectional size of the strip, it was found that the test data of the strip cross section were in good agreement with the theoretical value. Comparison of experimental and theoretical values of strip cross-section dimensions is shown in Figure 21. When the roll gap was increased to 0.5 mm, the dimensions of the strip cross section were found to be slightly larger than the theoretical values, and the experimental values and simulation results showed consistency. This indicates that when the roll gap is large, the strain concentration phenomenon is effectively alleviated and defects such that cracks do not occur, but it has a greater effect on the forming accuracy of the strip cross section.

**Figure 22:**
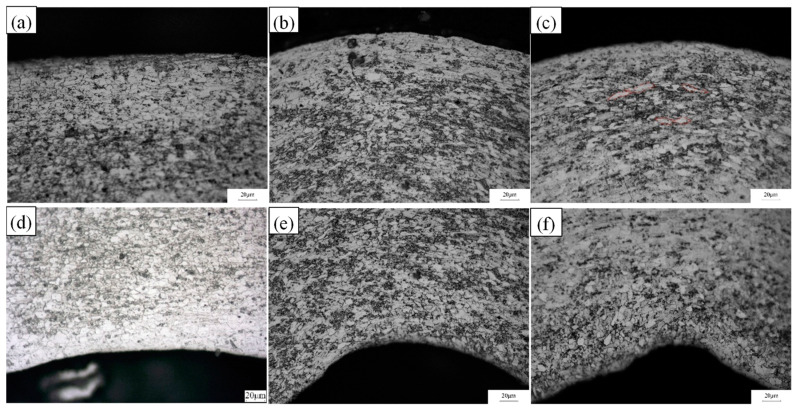
Microstructure variation of the inside and outside part of corner 2 for each pass at a roll gap of 0.4 mm. (**a**) Outside of corner 2 of pass 2. (**b**) Outside of corner 2 of pass 3. (**c**) Outside of corner 2 of pass 4. (**d**) Inside of corner 2 of pass 2. (**e**) Inside of corner 2 of pass 3. (**f**) Inside of corner 2 of pass 4.

The microstructure morphology of the strip after the second/third/fourth pass at corner 2 with the roll gap of 0.4 mm is shown in Figure 22, As the number of forming passes increased, the transverse tensile force on Corner 2 increased, causing the grains on its outer side to elongate gradually; by the fourth pass, the fibrillation of the grains became highly apparent. Nevertheless, the grains situated in the inside of corner 2 experienced a transformation from tension to compression while the forming angle decreased progressively, and this part of the grains showed extrusion characteristics (the inner side was mainly stressed by compressive stress): still isomorphic crystals, even finer crushed crystals, appeared on the edge of the inner region. The morphology of this feature corresponds to the stress analysis inside and outside the characteristic nodes in Section 3.1.2.

**Figure 23:**
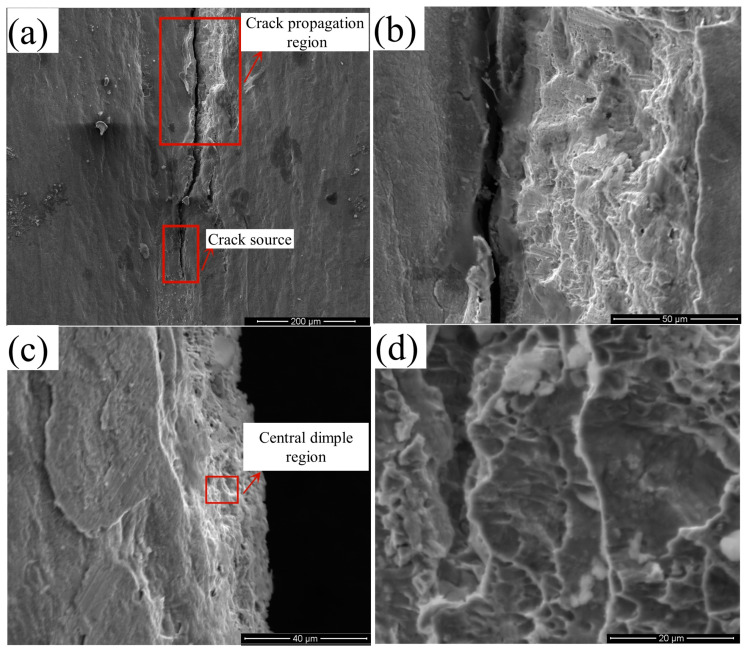
Morphological view of crack propagation at the top of corner 2. (**a**) Macroscopic morphology of crack source and crack extension region. (**b**) Enlarged view of the crack extension region. (**c**) Typical tough nest morphology of the mid-region (**d**) Enlarged view of the typical tough nest morphology.

The intermittent crack defects that occurred at the top of corner 2 were scanned and the microscopic morphology is shown in Figure 23. As can be seen from Figure 23a,b, once the crack was formed at the top of corner 2, the crack extension region showed a laminar tearing pattern and the layer-by-layer transition showed a step-like shape, which was very similar to a disintegrated fracture from the microscopic morphology, indicating that a relatively severe stress concentration phenomenon occurs at this location. At the early stage of crack initiation, the region of corner 2 was subjected to a large lateral stress from the roll gap, which provided the tangential stress of the laminar crack, resulting in stress concentration in this region, and strain was greater and crack propagation rate was extremely fast. Figure 23c shows that a typical tough nest morphology appeared when the laminar crack reached the mid-region, indicating that when the crack extended to a certain distance, its expansion started to slow gradually, and since the strip was a plastic material, a typical tough nest morphology started to appear in the slow region. A partial enlargement of its ligamentous fossa morphology is shown in Figure 23d. Thus, the mechanism of longitudinal crack propagation is mainly a composite form of cleavage fracture + ductile fracture, and once crack propagation occurs, it will rapidly form tearing propagation and produce destructive cracks as cold roll forming proceeds.

## 4. Conclusions

(1) This paper proposes a new technology for the on-rail cold forming of space truss rods. It was found by studying the effect on cold roll forming of asymmetric strip cross section under different roll gaps that the stresses on the strip during the forming process are mainly concentrated in corners 2/4/6, with the largest strain values in corner 2. As the number of passes increases, the maximum equivalent strain values are 0.09, 0.24 and 0.64 in succession when the roll gap is 0.3 mm. The equivalent roll gap variation of 0.4 mm/0.5 mm is relatively similar, and for corner 2, the equivalent variation is approximately 0.07/0.15/0.44 as the number of forming passes increases. The forces in the web plate region are dominated by elastic stresses, and little plastic deformation occurs. From the analysis of the stress–strain history of the characteristic nodes in corners 2/4/6, it can be seen that the stress and strain are mainly at the moment of action of the two roller wheel sets, the stress during the travel period is mainly dominated by the elastic stress, and there is no change in the strain. During the deformation process, the stress type of node 55786 shows two tensile types and one compressive type, and the stress types of nodes 48594 and 15928 show two compressive types and one tensile type, and the strains of all three nodes conform to the plane strain characteristics. Taking into account the stress–strain of the strip cross section and the precision of the strip dimensions, a roll gap of 0.4 mm is relatively good.
(2) As the roll spacing increases, the peak longitudinal stress of the strip passing through each roller group shows a small incremental trend, but the peak stress is around 380 MPa. When the roll spacing is large enough, it can effectively reduce the longitudinal strain, but it will lead to an increase in the longitudinal stress, which easily leads to the rebound of the strip and aggravate the phenomenon of strip edge wave. Combined with the fluctuation in strip edge at different roll spacings and factors such as manufacturing cost and volume, a roll spacing of 100 mm is appropriate.
(3) After experimental verification, the strip can be successfully prepared according to the cross-section requirements when the roll gap is 0.4 mm and the roll spacing is 100 mm. Observation of the microstructure of corner 2 shows that as the number of passes increases, the grains on the outside of corner 2 show obvious fibrosis characteristics, while the grains on the inside are extruded and still show an equiaxial shape. When the roll gap is reduced during strip formation, the strip edge is susceptible to edge waviness and the top of corner 2 is prone to intermittent cracking. Analysis of the cracking region of the strip has shown that the crack propagation mechanism is mainly based on cleavage fracture and ductile fracture.

## Figures and Tables

**Table 1:** Mechanical properties and chemical composition of Q195 galvanized steel strip.

Yield Strength/MPa	Tensile Strength/MPa	Elongation Rate/%	Quality Scores %							
			C	Si	Mn	S	P	N	Cr + Ni	Fe
403	441	27.5	0.12	0.3	0.5	0.035	0.035	0.012	0.1	Bal.

**Table 2:** Roll bending angle distribution for each pass.

Number of Forming Passes	1	2	3	4
Corner 2-1 (partition 2-1)	0°	20°	40°	65°
The increment Δθ of corner 2-1	0°	20°	20°	25°
Corner 2-2 (partition 2-2)	0°	20°	40°	55°
The increment Δθ of corner 2-2	0°	20°	20°	15°
Corner 4 (partition 4)	0°	20°	40°	55°
The increment Δθ of corner 4	0°	20°	20°	15°
Corner 6 (partition 6)	0°	30°	60°	90°
The increment Δθ of corner 6	0°	30°	30°	30°

**Table 3:** Experimental scheme of strip simulation process.

Simulation Parameter	1	2	3
The gap between the rolls	0.3 mm	0.4 mm	0.5 mm
The spacing of the rolls	100 mm	120 mm	140 mm

## Data Availability

The data presented in this study are available on request from the corresponding author.
